# Traversing through the Mechanistic Event Analysis in IL-6 and IL-17 Signaling for a New Therapeutic Paradigm in NSCLC

**DOI:** 10.3390/ijms25021216

**Published:** 2024-01-19

**Authors:** Riya Khilwani, Shailza Singh

**Affiliations:** Systems Medicine Laboratory, National Centre for Cell Science, NCCS Complex, Ganeshkhind, SPPU Campus, Pune 411007, India; riyakhilwani@nccs.res.in

**Keywords:** IL-6, IL-17, NSCLC, NFkB, M2 macrophages, systems immunology

## Abstract

IL-6 and IL-17 are paradoxical cytokines that progress inflammatory states in chronic diseases, including cancer. In lung cancer, their role has been elucidated to favor cancer development by modulating signaling mechanisms critical to cellular growth. The intrinsic ability of these cytokines to influence macroautophagy is yet another reason to facilitate lung cancer. Here, we employed a systems immunology approach to discover the mechanistic role of these cytokines in cancer development. In a biological system, at later stages, the activation of NFkB stimulates immunosuppressive phenotypes to achieve tolerating effects in a transformed cell. We found that the upregulation of cytokines signaled M2 macrophages to modulate tumor responses through the activation of autophagic intermediates and inflammasome mediators. This caused immune perturbations in the tumor microenvironment, which were associated with cancer inflammation. To address these inflammatory states, we performed triggered event analysis to examine whether overexpressing immune effectors or downregulating immune suppressors may have an effect on cancer reversal. Interestingly, the inhibition of immune regulators opposed the model outcome to an increased immune response. Therefore, IL6-IL17-mediated regulation of lung cancer may address tumor malignancy and potentiate the development of newer therapeutics for NSCLC.

## 1. Introduction

Being a more prevalent form of lung cancer, NSCLC (non-small cell lung cancer) accounts for 80–85% of the significant death rates across the globe. Over the years, due to the availability of targeted therapies and newer treatment approaches, there has been an improvement in NSCLC outcomes. However, the majority of cases seem to be progressing because of the increased resistance to anti-cancer drugs. This implies that there are other host factors that play a major role in shaping the pathophysiological conditions during NSCLC progression [1]. Cytokines are soluble factors that simulate biological reactions in response to internal and external stress. On the basis of their effect on host cells, cytokines are either pro-inflammatory or anti-inflammatory in nature. In the context of lung cancer, pro-inflammatory cytokines act to promote the infiltration of those subsets that have an anti-tumor effect and are termed tumor suppressors. However, anti-inflammatory cytokines possess a pro-tumor effect, where they help immune suppressors aggravate cancer transformation [2]. Additionally, there are several cytokines that behave both as pro- and anti-inflammatory cytokines and generate a pleiotropic effect during the advancing stage of cancer. Indeed, these molecules co-stimulate both the effector cells and the diseased phenotype and thus increase the susceptibility of healthy cells to tumor antigens. Over the list of cytokines, IL-6 and IL-17 tend to hype inflammatory responses and remodel lung cancer pathogenesis through immunological defense mechanisms. Due to the specificity of these cytokines to potentiate cancer development, IL-6 and IL-17 drive oncogenesis by regulating several signaling mechanisms important to tumor growth and inflammation [3,4,5]. At higher concentrations, these are likely to trigger and deregulate autophagy mechanisms [6,7], which has an indirect effect on cancer development. Since their role is to favor tumor growth, designing a novel inhibitor or blocking autophagy intermediates would help regress NSCLC development [8].

### 1.1. IL-6 and Signaling Events

**Figure 1:**
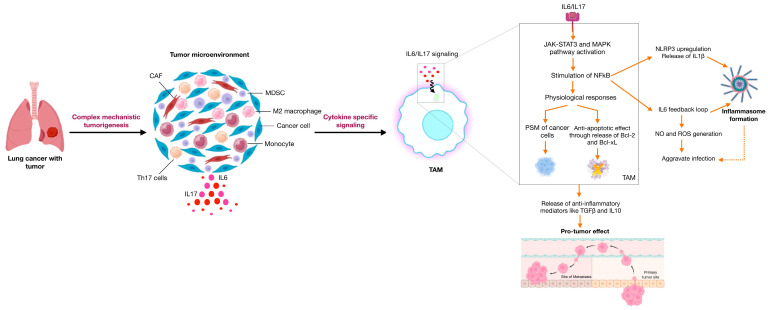
Schematic representation of IL-6 and IL-17 signaling in lung cancer development: Tumor development in the pulmonary state begins with the small tumor mass that signals other immune cells to establish TME. The immune pool at the transformed site releases cytokines like IL-6 and IL-17, which activate TAM to facilitate cancer inflammation through the formation of an inflammasome complex.

With its advent in 1986, IL-6 emerged as a pleiotropic cytokine [9] to be involved in regulating several biological mechanisms, including developmental processes [10], immune networking [11], homeostatic equilibrium [12], and innate responses [13]. Being a classical member of the IL-6 cytokine family, it was originally discovered as BSF-2 to elicit antibody production and control acute phase protein generation [14] It has also evolved as a key player in modulating cancer cells through its transactivation via STAT3 mechanisms, which constitutively express IL-6 and other chemokines important to the disease phenotype [15]. Through its ability to upregulate NFkB, it facilitates autophagic events to further chronic inflammation (refer to Figure 1). In lung cancer, IL-6 has an expanding role in inducing the expression of cell cycle proteins [16]. In a recent study by Weber et al., the role of IL-6 has been reported to mediate the activation of the immune suppressor MDSC, which inhibits the activation and tumor suppressive potential of NK cells and T cells [17]. Integrating the above findings, the multivariate role of IL-6 both in disease pathophysiology and immunotherapeutics is therefore clear.

#### 1.1.1. The Importance of IL-6 Mediated Regulation in the PSM of Cancer Cells

The notion that cytokines drive tumor development is well evidenced. There are certain molecules that link the innate and adaptive immune systems in order to regulate cellular events underlying cancer progression. Among the list, the overexpression of IL-6 has been associated with the onset of signaling cascades in lung cancer, breast cancer, prostate cancer, and other human cancers. The intrinsic ability of IL-6 induces tumor-activating pathways such as JAK/STAT, Ras/MAPK, and PI3K/AKT, which together culminate in stabilizing the anti-inflammatory subsets in the TME. As a result, the production of immunomodulatory molecules favors tumor development [18]. Apart from regulating cancer pathogenesis, it is involved in delaying apoptotic but triggering autophagic mechanisms that promote the survivability of inflamed cancer cells. Nevertheless, the necrose cells undergo molecular transitions to attain a malignant phenotype. These genetic alterations favor the tumor metabolic process and enhance the proliferative and migratory features of lung cancer cells. All the events together function to resist cancer cell death through evading growth suppressors, metabolic remodeling, and the impairment of the DNA repair processes [19].

#### 1.1.2. IL-6 Upregulates NFkB to Address Chronic Inflammation

IL-6 and the NFkB signaling axis connect chronic inflammation to lung cancer. It is believed to be a potent pleiotropic molecule involved in facilitating neoplastic transformation [3]. In lung cancer, IL-6 is activated through different signaling mechanisms that work in a context-dependent manner. During cancer initiation, IL-6 functions to proliferate CD8 cells to release cytolytic molecules and maintain lung homeostasis. However, during later stages, IL-6 regulates IL-10 production [20] by favoring the physiological role of inflammatory subsets like MDSC. Tumor-specific M2 macrophages act as a convergent immune polarizing cell of autophagy in NSCLC [8]. IL-6 secreted through different signaling mechanisms converges to bind to the IL-6R in macrophage population [21]. This results in the activation of the master regulator, NFkB, which takes charge of producing tumor proliferative signals and building a reducing host environment. Through its potential to upregulate Nlrp3 expression, IL-6 functions to assemble an inflammasome complex to drive chronic inflammation [22]. This is mediated through the production of mature inflammatory mediators like IL-1β and IL-18 that have a long-term effect on inflamed lung cancer cells.

### 1.2. IL-17 and Mitogenic Signals

IL-17 is a pro-inflammatory cytokine that is produced by Th17 cells expressing the RORγt receptor and generates an anti-tumor response during the initial stages of cancer. It plays a biological role in the recruitment of immune cells like macrophages and neutrophils through inducing related cytokines [4]. The overexpression of IL-17 is associated with the onset of many diseases, including cancer [23], autoimmunity [24], bacterial infection [25], and allergy [26]. Previous literature has shed light on the fact that IL-17-mediated chronic inflammation promotes the tumorigenic potential of lung cancer cells [5]. Recalling its effector functions, IL-17 influences cancer cell survival by polarizing macrophages, regulating the expansion and proliferation of anti-inflammatory subsets, promoting the EMT process, and releasing pro-angiogenic factors. During the initial stages, IL-17 has a pronounced effect on transducing tumor cells to activate STAT3 signaling [27], along with its downstream effectors such as NF-κB and anti- apoptotic proteins. As a result, they acquire an enhanced ability for self-induction, which confers them resistance against apoptotic signals.

#### 1.2.1. Role of IL-17 in Enhancing Lung Cancer Susceptibility

Certain factors, including smoking, are considered important in the process of causing IL-17 gene polymorphism. This genome variation has implications in the aspect of tumor development, where the upregulation of IL-17 isoforms has been shown to enhance the tumorigenic potential of lung cancer cells [28]. The data from Liao et al. has shown that the deep mutations in IL-17A, IL-17E, and IL-17F are linked to cancer progression [29]. Upon cancer transformation, pulmonary IL-17 expression increases manifold, which has a significant effect on the upregulation of those proteins that are associated with tissue remodeling [30]. These changes in the gene expression profile influence tumor cells under development to undergo subsequent proliferation and attain metastatic potential.

#### 1.2.2. IL-17 Shapes the TME

As discussed about the role of IL-17 in tumor development, its inherent ability to facilitate chronic inflammation makes it a key regulator in NCSLC progression. It is shown to have a paradoxical role during lung cancer development. During the initial stages, it reflects an anti-tumor effect; however, during later stages, it progresses cancer through modulations in the tumor microenvironment. Indeed, there is evidence suggesting their role in the development of new micro-vessels to support cellular life and promote the process of angiogenesis in the pulmonary environment. As mentioned about the significance of STAT3 in maintaining the IL-17 feedback loop, it also functions to enhance the migration ability of human lung adenocarcinoma cancer cell lines like A549 and H1299 [31]. The activation of transcription factors, therefore, drives the EMT process and induces the expression of NLRP3 to remodel the TME. It not only modulates the angiogenic potential of tumor cells but also produces immune effectors to support the host’s lung inflammatory state (Figure 1). This implies that the survival effect of cells in a hypoxic tumor environment is the result of immune infiltration, like macrophages, into the tumor sites [32].

### 1.3. Cellular Crosstalk and Cytokine Implications in Tumor Development

The TME in lung cancer is composed of different immune subsets, including CAFs, monocytes, NK cells, DC, MDSC, T cells, and M2 macrophages, along with the transformed pulmonary cell. All the cells together constitute a complex signaling network important to cancer development. In lung cancer, the process of oncogenesis begins with the binding of LPS to a TLR4 receptor, which initiates different signaling events to address a cancerous phenotype [33]. Of all the activated signaling cascades, the activation of Myd88 [34] allows the stimulation of NFkB [35], which has a significant effect on transcribing the PIC gene specific to lung cancer. However, the activation of Erk signaling through Myd88 intermediates [36] stimulates AP1 transcriptional activity to present a combined effect on cancer-specific gene expression. Similar to LPS signaling, the activation of the IL-1β pathway signals tumor cells to express immune mediators like IL-6 [19], TNFα [37], CCL2 [38], VEGF [39], PDGF-b [40], and MCSF [41]. PDGF-b, thus produced through pulmonary cells, stimulates PI3K/Akt/mTOR/STAT3 in [42] to generate VEGF. VEGF, being an angiogenic factor, furthers the process of angiogenesis in hypoxic lung cells. In addition to PDGF-b signaling, TNFα and IL-6 signaling facilitate NFkB and JAK-STAT3 activation, respectively. All these signaling events work to regulate inflammatory diseases, including cancer [43]. Also, IFNγ and IL-17 translocated from T cells activate CAFs through the PI3K and MAPK signaling pathways in order to enhance AP1 transcriptional regulation. The aforementioned signaling event favors the transcription of survival genes to produce Cyclin D1, Bcl-2, and Bcl-xL [44,45] and inflammatory genes to influence IL-6 and IL-4/13 production.

Monocytes contribute to cancer cell survival through different signaling ligands. CCL2 produced through TC [46] polarizes macrophages into the M1 phenotype through IL-1β and IL-23. Alongside, IFNγ from T cells and NK cells converge STAT3 mechanisms to produce IL-6. The production of IL-23 and IL-17 by CD4+ T cells employs NFkB activation to generate IL-6 cytokines for cell proliferation. Furthermore, the reciprocal induction of DC and NK cells regulates cancer cell survival through the activation of STAT molecules for IFNγ and IL-12 secretion. The initiation of the above-described signaling events is responsible for the progression of cancer inflammation in a compromised host. The onset of the immune-tolerating mechanism produced through MDSC significantly addresses cancer outcomes. This is achieved through the upregulation of immune modulatory molecules like IL-10, iNOS, and arginase through an additive effect of STAT1 and STAT6. Apart from regulatory cells that influence uncontrolled proliferation, T cells have an innate potential to decide cancer cell fate. At stages, it signals through IL-23 to generate enormous IFNγ levels. The differentiation of CD4+ Th17 cells is mediated through IL-6, which activates STAT3 signaling to upregulate the RORγt transcription factor. At later stages, the polarization of CD4+ into Th2 cells and Treg is important in modulating an immune condition [47]. This is possible through IL-4/IL-13 signaling and STAT6 activation to maintain GATA3 gene expression in a reduced environment [48]. All the signaling elements discussed above show a context-specific role in tumor survival and development.

***M2 macrophages mediating macroautophagy and inflammasome***

**Figure 2:**
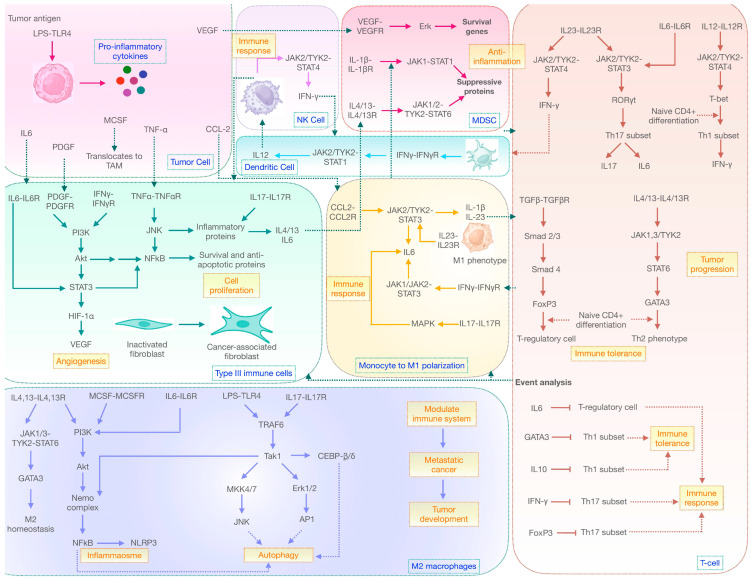
Schematic model representation of the IL6-IL17 signaling axis with autophagy in NSCLC.

Until now, we have discussed how cytokines play an important role in immune polarization at different stages of cancer. Of all the immune cells studied, M2 macrophages are critical to cancer survival and development because of their two major roles: Firstly, they facilitate the formation of an inflammasome complex that is essential to the chronic state of cancer [49]. Secondly, it overexpresses autophagy genes to sustain nutrient- deprived cancer cells and generates a macroautophagic response [50]. This is brought about by the signaling cascade activated through different cytokines. The initiation of IL-17 and MCSF drives NFkB to upregulate the expression of the Nlrp3 gene to drive cancer proliferation [22]. Through its inherent ability, it produces mature forms of IL-1β and IL-18 to achieve chronic inflammatory states. The increasing survivability of cancer cells in a redox environment elicits the immunosuppressive potential of TAMs, which can be further supported by other signaling events. In order to support M2 homeostasis, IL-4/13 cytokines help activate STAT6 signaling. Also, LPS and IL-6 favor the process through MAPK and PI3K signaling. This results in the activation of transcription factors like NFkB, AP1, JNK, Erk, and CEBP-β/δ, which act to regulate autophagy through the expression of LC3II, Beclin-1, ATG5, ATG12, and other proteins. The overall result is an increased autophagy response and redox homeostasis in lung cancer cells (Figure 2). Therefore, therapeutically targeting these mediators will help to resolve cancer outcomes in cancer patients.

Since the role of IL-6 and IL-17 has been shown to activate multiple signaling pathways important for cancer development, the differentiation of anti-inflammatory subsets is responsible for stabilizing the phenotype of tumor suppressors. Therefore, the above introduction concludes that IL-6 and IL-17 are linked with poor prognosis in lung cancer patients. Insilico approaches to specifically targeting the downstream effectors of IL-6 and IL-17 signaling pathways would help to address the immune-suppressive environment in the TME of pulmonary cells.

## 2. Results

### 2.1. Reconstructed Mathematical Model and Simulation

**Figure 3:**
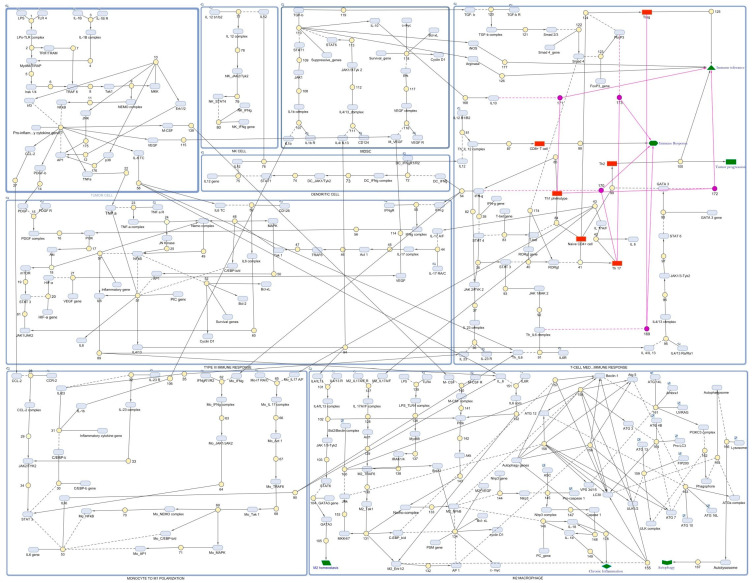
The reconstruction of a mathematical model by employing the IL-6/IL-17 signaling axis and its effect on autophagy resulting in chronic inflammation in lung cancer: In a figure, a rectangle indicates eight different compartments. A light-blue oval shape represents species. The yellow color indicates the reactions involved. The red color indicates those species that take part in events. A pink circle indicates triggered events, and a green color represents the immune state of the cell. Here, (**a**) TC: releases different cytokines to initiate cancer signaling; (**b**) NK cells: play a role in generating protective immune responses during the initial stages of cancer; (**c**) MDSC: an immune-suppressive phenotype that progresses tumor development by generating redox homeostasis; (**d**) DC: similar to NK, it generates an effector response against cancer antigens; (**e**) Type III immune response: a specialized phenotype in cancer that supports cancer survival and development; (**f**) monocyte-to-M1 polarization: monocytes function as effector cells by differentiating into the M1 macrophage phenotype and releasing pro-inflammatory molecules; (**g**) T-cell-mediated immune response: predominant regulator of an immune response in cancer; and (**h**) M2 macrophages: facilitate tumor development through inducing macroautophagy, leading to chronic inflammation.

Since we have discussed the complex behavior of IL-6/IL-17 cytokines, it is, therefore, important to discern their integrated role in cellular mechanisms. In our study, the reconstruction of the mathematical model was conducted by considering different immune cells (Figure 3) that supported the cancer phenotype in a reduced environment. Here, the model comprised a total of eight compartments: TC, Type III immune cells (including CAFs, endothelial cells, and stromal cells), monocytes to M1 polarization, MDSC, NK cells, DC, T-cell mediated immune response, and M2 macrophages to understand the intricacies of signaling events in macroautophagy induction and inflammatory responses. After mathematical modeling, the model was simulated for 100 s (a time unit) to elucidate the immune network in tumor progression. In order to understand cancer cell behavior, the desired graph was obtained in a concentration vs. time manner. The model was built by considering a scenario where the production of different cytokines in a lung TME has an effect on the developing cancer mass. Refer to Table 1 for an overview of the parameters involved in reconstructing the mathematical model. Reactions that were incorporated for model building are represented in Supplementary Data S1.

Through the reconstructed model, we illustrated that the crosstalk between immune cells in the lung cancer model is a result of the IL-6/IL-17 signaling axis that is activated upon the recognition of tumor antigens by lung cancer cells. This activated the transcription of pro-inflammatory cytokine genes that express cancer-specific effectors to nurture the hypoxic pulmonary tissues. The molecules thus produced stimulated other immune subsets that acted simultaneously to support tumor survival. We observed that when the inflammation persisted, the cytokines produced through TC polarized M0 macrophages into the M2 phenotype to achieve redox homeostasis in reduced cellular conditions. This elicited TAMs to overexpress those genes that are essential to driving stress in the TME. Since the starved cancer cells necessitate improved blood supply, the expression of NFkB and other transcription factors at later stages signal autophagy genes in order to nourish the starved cells. This resulted in the macroautophagic response in M2 macrophages, which makes sense given that under nutrient-deprived conditions, macroautophagy resisted cancer cell death.

As per the previous study, it has been reported that the mediated inflammatory responses acknowledge the survivability of cancer cells [51]. Also, in our study, we noticed that few cytokines, when acting in an autocrine fashion, overexpressed NFkB to facilitate chronic inflammation through IL-1β and IL-18. This indicates that upon lung cell transformation, the crosstalk between immune subsets favors cancer development through modulating autophagic and inflammatory responses. Apart from its role in progressing cancer inflammation, IL-6 and IL-17 play a critical role in providing the host with a protective immune response against pathogens. Its role in infections, including bacterial, viral, and fungal, has been shown to activate the signaling mechanisms to regulate the migratory and effector functions of immune cells [25,52,53]. This implies that during the early stages of infection and inflammation, IL-6 and IL-17 generate a protective response. However, as the inflammation progresses, both of these cytokines work synergistically to progress inflammatory events in cancer.

**Figure 4:**
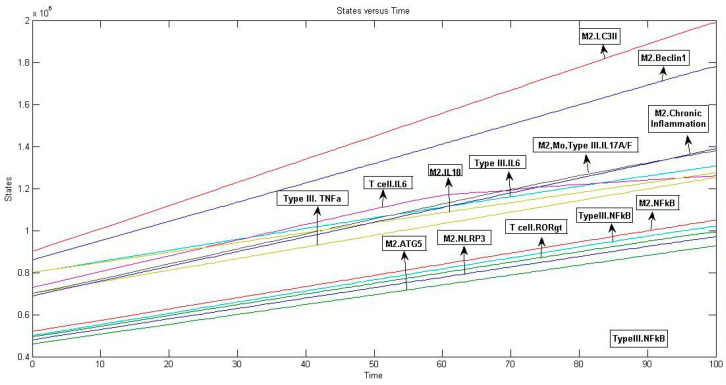
Simulated lung cancer mathematical model: The figure highlights those components that had the highest concentration across other elements and is arranged in decreasing order of their concentration. These components include cytokines like IL-6, IL-17, IL-18, and TNFα; autophagic proteins like LC3II, Beclin-I, and ATG5; transcription factors, NFkB and RORγt; inflammasome complex, NLRP3; and chronic inflammation state. Graph represents: M2.LC3II, M2.Beclin-1, M2. Chronic inflammation, M2, Mo, Type III.IL-17A/F, Type III.IL-6, M2.IL-18, T cell. IL-6 Type III.TNFα, M2.NFkB, Type III.NFkB, T cell.RORγt, M2.NLRP3, M2.ATG5.

We simulated the model and observed that the major output after simulation was the gene products that conferred autophagy resistance and facilitated chronic lung cancer inflammation (Figure 4 and Supplementary Data S2). For all the derived components, we considered the top 15 hits and interpreted the final model outcome. We found that autophagy genes like LC3II, Beclin-1, and ATG5 had the highest concentrations. Also, IL-6, IL-17, and TNFα were found at higher levels, which suggest that cytokines act in parallel to modulate immune responses in cancer. The expression of RORγt was expected to be higher to proliferate CD4+ Th17 cells for IL-17 production. Additionally, the expression of NFkB stimulated Nlrp3 gene expression to establish lung cancer inflammation. Altogether, these data suggest that the activation of IL-6/IL-17 signaling events increases disease severity, which leads to chronic inflammation and autophagy.

### 2.2. Triggered Event and Analysis

As we talked about the application of triggered events to tracking immune alterations, we applied events to check if the modulations in the simulation of effector molecules would result in changes in the cancer-specific immune response. Triggered events and analysis for all reactions are summarized in Table 1.

In event analysis, TC secreted IL-6 inhibited the immunomodulatory T-regulatory cell to overcome the effect of immune tolerance and drive pro-inflammatory responses in an inflamed site. Similarly, IFNγ and FoxP3 had an inhibitory effect on Th17 cells producing the IL-17 cytokine. Since IL-17 overexpression is linked to an exaggerated immune response and increases cell susceptibility to cancer, the inhibition of IL-17 may have a surprising effect on cancer resolution. As expected, we observed that inhibiting IL-17 through IFNγ and FoxP3 reversed the immune-tolerating effect and facilitated immune responses in a cell. In contrast, GATA3 and IL-10 dominated to suppress the Th1 phenotype toward cancer progression. This modulated the host’s responsive genes and enhanced the phenomenon of immune tolerance. This reveals that the production of pro- inflammatory cytokines in cancer may help the developing tumor mass escape immune perturbations.

Similarly, in a biological host, the pro-tumor effect can be enhanced by modulating the cytokine expression profile by either designing a specific drug or an inhibitor. According to our model, the inflammatory states can be resolved either by overexpressing IL-6, IFNγ, and FoxP3 or by reducing the cellular levels of GATA3 and IL-10 molecules. Alternatively, it can be rescued by designing peptides to stop CD4+ Th17 and T-reg differentiation. Together, the above findings confirm that reducing the effect of anti-inflammatory subsets can help bring immune homeostasis back to normal [54].

### 2.3. Principal Components and Analysis to Discern Crucial Elements

**Figure 5:**
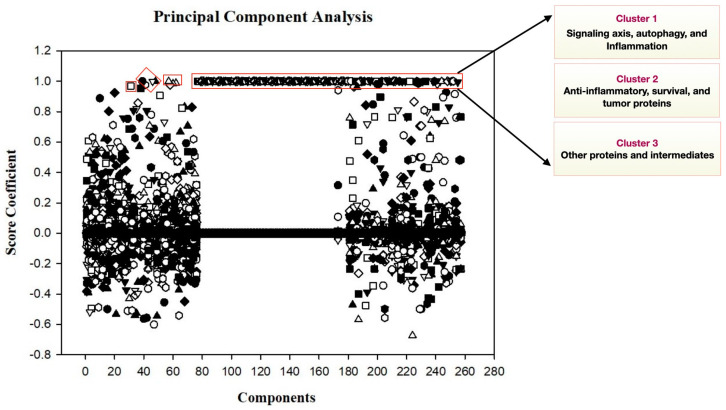
Principal component analysis to derive crucial elements: The figure highlights those elements that had a PC score of 1.0, which was considered to extend further analysis. Here, clusters include, Cluster 1: IL-6, IL17, autophagy and inflammasome intermediates; Cluster 2: IL4, IL13, Bcl-2, Bcl-xL, Hif-α, MCSF, and Cyclin D1; Cluster3: STAT proteins, pro-inflammatory cytokines, and signaling proteins.

By using the sensitivity scores (Supplementary Data S3), the principal component analysis was performed to derive the critical and most sensitive components in the interconnected signaling pathways. As a result, we obtained many molecules that majorly specified the model and were categorized into three different clusters to minimize data loss. Cluster 1 included all those components that were important to the signaling axis, autophagy, and inflammation. Cluster 2 had anti-inflammatory, survival, and tumorigenic factors. Cluster 3 included all those intermediates that mediated the signaling pathways in between (Figure 5). The Supplementary Data S4, includes all those components that were included in three different clusters. PC analysis reflects that precisely targeting autophagy and inflammasome mediators may help to disintegrate the signaling network and overcome malignancy via the downregulation of survival genes.

### 2.4. Flux Balance Analysis

By performing flux balance analysis in a lung cancer model, we got to know that there are few metabolic reactions that have a crucial effect on defining the cancer phenotype. Out of the total reactions that were included in the study, we considered those reactions that had a flux in the range between 7550 and 1050. By doing so, we obtained the top 58 reactions that had a higher flux and were hence considered important for further analysis. The flux for all the reactions was calculated in terms of molecules per second (mol/s).

In order to understand the major network contributors, we analyzed the top 10 reactions (illustrated in Table 2). Interestingly, we found that the reaction involving the differentiation of CD4+ Th17 cells to produce IL-6 and IL-17 had the highest flux, which coincides with the above result that IL-6 and IL-17 are important to initiate the signaling cascade. Additionally, the other nine reactions indicated the onset of inflammatory responses through the generation of immunomodulatory molecules. Hence, targeting the top candidates would reverse the model phenotype toward a healthy phenotype. The final flux for all the reactions is represented in Supplementary Data S5.

### 2.5. Model Reduction

**Figure 6:**
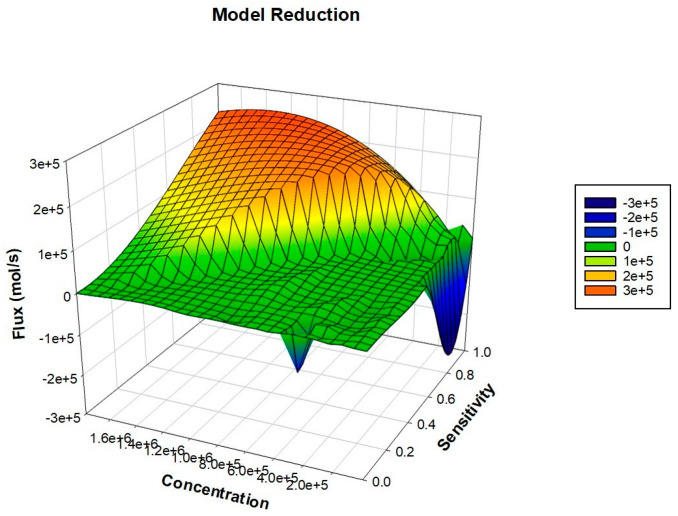
A graphic representation of a reduced model of lung cancer: The dome shape at the top includes the most sensitive components important to regulating cancer survival and development. Here, the *X*-axis represents component-sensitive scores, the *Y*-axis represents molecular concentration, and the *Z*-axis signifies the flux of a particular reaction.

In order to reduce the model dimensionality, we performed MR by combining the results for flux balance and sensitivity analysis (Supplementary Data S6). At the end, we obtained a quasi-potential landscape (a 3D mesh network), which included all important reactions sufficient to simulate the reconstructed model (Figure 6). The dome-shaped structure in the result signifies the region that includes higher flux reactions and the principal components. Moving down the peak, we found that there were elements that were least sensitive and also had lower flux. Therefore, to exclude less important components, we reduced the model by 66.85%, meaning that 33.15% of the reactions significantly contributed to the final model outcome.

### 2.6. Network Crosstalk Score Analysis

The network crosstalk score compares an individual pathway in the model system to the entire network in order to investigate which element in the biological network is affecting other components the most.

Since there were many components that showed a cross-connection across the model system, we decided to rule out the crosstalk score for all eight compartments. After analyzing the entire result, we decided on a cut-off score of 4 and interpreted the result accordingly (Supplementary Data S7). Considering the same fact, we observed that, despite playing an important role during cancer inflammation, cells like tumor cells, NK cells, DC, and MDSC did not show higher interconnections and, hence, were not regarded in the analysis. However, we did observe crosstalk points for Type III immune response, monocytes to M1 polarization, T-cell-mediated immune response, and M2 macrophages. The results for the same are attached below.

**Figure 7:**
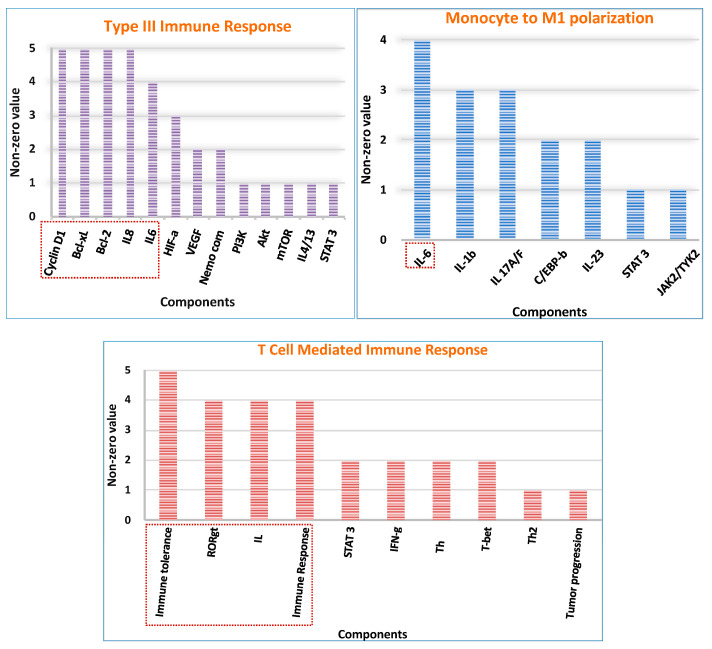

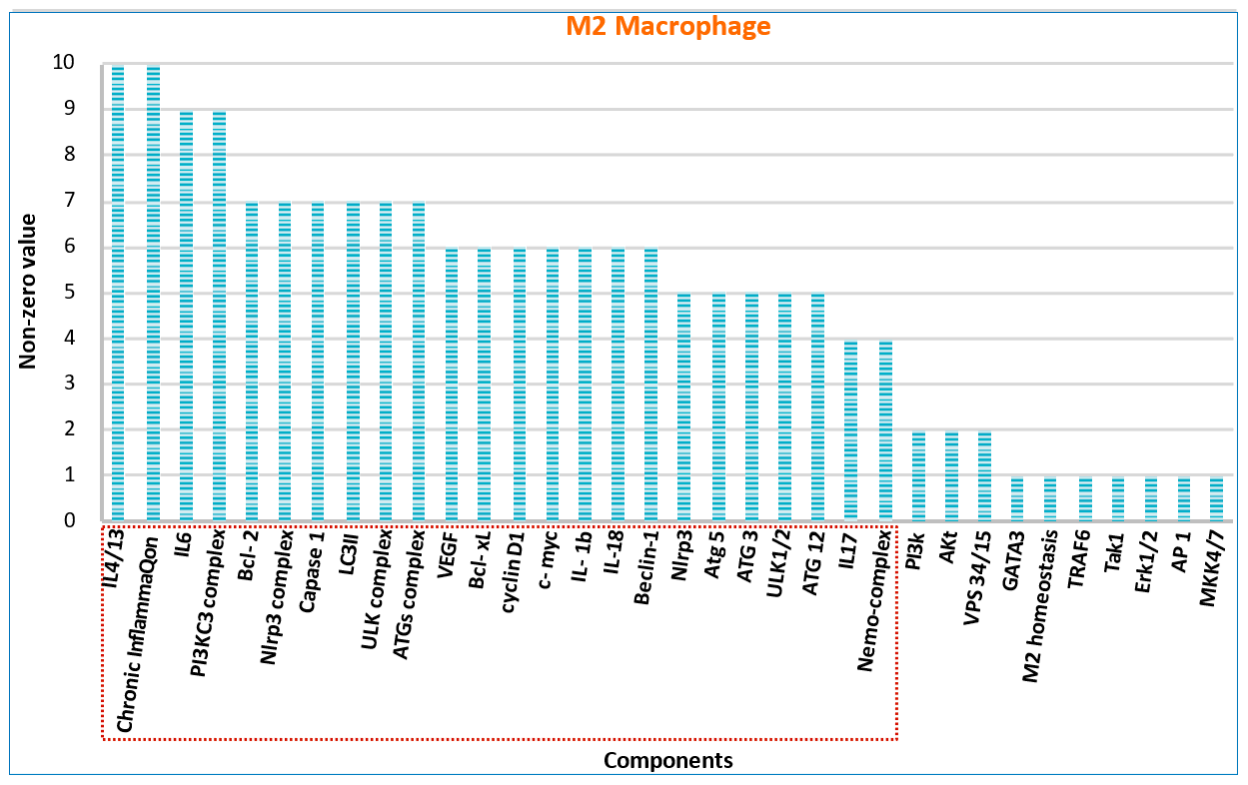
Crosstalk score analysis for different compartments: The scores were calculated for all eight compartments, but those who had a significant score greater than 4 were considered. The red boxes in different compartments signify important elements of the network.

In Type III immune response, survival genes like cyclin D1, Bcl-2, and Bcl-xL had a cross- talk score of 5, and IL-6 had a score of 4. In monocytes to M1 polarization, IL-6 had a higher score of 4, followed by IL-17. The T-cell compartment showed a higher score for immune tolerance, which is 5, whereas RORγt, IL-6, and the immune response showed a score of 4. In accordance with the above findings, M2 macrophages showed significant variations and the highest number of crosstalk points. Here, anti-inflammatory cytokines and chronic inflammation had a score of 10. IL-6 and autophagy intermediate, which is PI3KC3, reflected a score of 9. We observed 7 scores for anti-apoptotic protein (Bcl-2), NLRP3 complex, inflammatory factor (Caspase1), and autophagy intermediates like LC3II, ULK complex, and ATG complex. Also, angiogenic factor (VEGF), survival proteins (Bcl-xL, cyclin-D1, and c-myc), inflammatory mediators (IL-1β and IL-18), and autophagy protein (Beclin-1) had a score of 6. Proteins related to inflammasomes and autophagy showed a score of 5. However, IL-17 and the NEMO complex had the lowest score among all the entities, which was 4 (Figure 7).

Theoretically, to lay down the above analysis, it is needed to recall the signaling pathways and their plausible effect on lung cancer. As discussed, IL-6 and IL-17 can have a pleiotropic effect; they display a paradoxical role in cancer, which is context-dependent. In the early stages, IL-6 and IL-17 work to elevate a pro-tumor effect by stabilizing the CD4+ Th17 phenotype. However, at later stages, it helps cancer cells provide energy substrates through autophagic intermediates, resulting in chronic inflammation. Taken together, we can infer that both IL-6 and IL-17 can have a significant effect on supporting cancer cell survival during later stages of development.

## 3. Discussion

Because it is a leading cause of concern in today’s era, lung cancer reports the highest number of deaths worldwide. Among the subtypes of lung cancer, NSCLC accounts for more than 85% of the cases. It is a major subtype that affects both smokers and non-smokers, and its treatment varies depending on the stage and location of the tumor. Although the availability of cancer drugs has been reported to address disease severity, the emergence of multi-drug resistance has reduced the survival rate of cancer patients. Being the deadliest disease with no possible cure available, it urges us to understand the mechanistic regulation underlying tumor escape and development. In order to address an unresolved issue, one needs to employ advanced networking strategies to track actionable nodes that remodel cellular homeostasis by regulating different biological mechanisms. To solve this in the initial stages, it requires the integration of systems biology to improve the overall survival rates and cure rates of lung cancer patients. In our study, we employed computational approaches to reconstruct a mathematical model so as to gain insights into the overexpressed components during lung inflammation. As expected, we identified transcription factors, autophagic proteins, and inflammasome mediators as the most sensitive elements. This analysis revealed that these species may serve as an important cancer target, and targeting them with a specific peptide, drug, or inhibitor may help to cure the root cause of a disease, irrespective of the tumor stage. Therefore, achieving the ultimate cure to treat lung cancer in an early metastatic state or at later stages will really be a great milestone in the field of cancer immunotherapeutics.

IL-6 and IL-17 cytokines, as discussed, play a role in the survival and proliferation of transformed cells; it is, therefore, necessary to restrict their downstream effectors in order to abolish mitogenic signals in the TME. In lung cancer, IL-6 during the initial stages worked to generate an anti-tumor effect along with other cytokines that were secreted in the tumor microenvironment. It is important to note that tumor cells at any stage of development release anti-inflammatory molecules to mark immune-tolerant effects in lung tissues. Since IL-6 acts through a positive feedback mechanism, it modulates IL-6 toward a pro-tumor effect-generating cytokine. This favors the translocation of IL-6 to other cellular environments, where it polarizes macrophages into an M2 phenotype. Being an important player in regulating immune signaling, TAMs offer the host a myriad of angiogenic factors that positively impact tumor progression. All these factors that pose an opposing effect are almost mediated through the activation of NFkB along with other transcription factors. Being a critical regulator of the inflammatory process, NFkB signals the formation of an inflammasome complex and establishes inflammation through mature end products like IL-1β and IL-18. Simultaneously, the signaling mechanism bifurcates to activate autophagy genes in order to prolong survival effects in cancer. As a result, the overexpression of inflammatory products along with autophagic intermediates enhances metastatic ability, which was previously dictated by a mathematical model.

Similar to IL-6, IL-17 behaves paradoxically to tailor tumor cells during oncogenesis. Unlike IL-6, IL-17 is produced by IL-6 and IL-23 signaling in CD4+Th17 cells. IL-23 is a pro- inflammatory cytokine that is critical to establishing the differentiation of CD4+ T cells into a Th17 subtype. Being predominant cells against bacterial and fungal pathogens at mucosal barriers, including the lung, CD4+ Th17 cells are highly susceptible to HIV-1 infection [55]. In lung cancer, IL-6 and IL-17 upregulate RORγt through STAT3 signaling mechanisms in order to produce IL-17 isoforms, IL-17A/F. This allows IL-17 to activate mitogenic signaling in Type III immune cells (cancer-associated fibroblasts, stromal cells, and endothelial cells). The resulting effect is the proliferation of cancer cells and the production of IL-6, anti-apoptotic factors, and other tumorigenic effectors. Focusing on the tumor-specific role of IL-17 in cancer, it also signals TAMs to generate an additive effect on IL-6 function. As dictated earlier, IL-6 activates STAT3 signaling to help cancer cells achieve their proliferative potential. According to previous literature, it has been shown that IL-17 can promote tumor growth through an IL-6-STAT3 signaling pathway. Here, after obtaining STAT3 as a principal component in the biological model, we could infer that the IL17-IL6-STAT3 signaling axis, through NFkB activation, is involved in achieving the malignant state of pulmonary cells. In M2 macrophages, the stimulation of the MAPK pathway activates a plethora of transcription factors, all of which have a defined role in establishing malignant states. The activation of JNK dissociates the Bcl-2-Beclin-1 complex, which frees Beclin-1 from inhibition and activates autophagy to sustain the nutrient-deprived tumor cells. On the other hand, the activation of NFkB, Erk1/2, CEBP-β/δ, and AP1 converges to activate autophagy genes. This results in the activation of canonical cell death mechanisms that serve to regulate disease severity. As mentioned above, the role of NFkB is important to drive inflammatory events and influence inflammation. Having said that, it is of paramount importance to dissect the signaling axis and the crosstalk between physiological defense mechanisms. This apparently helps to find cancer-specific targets in order to overcome uncontrolled malignancy.

**Figure 8:**
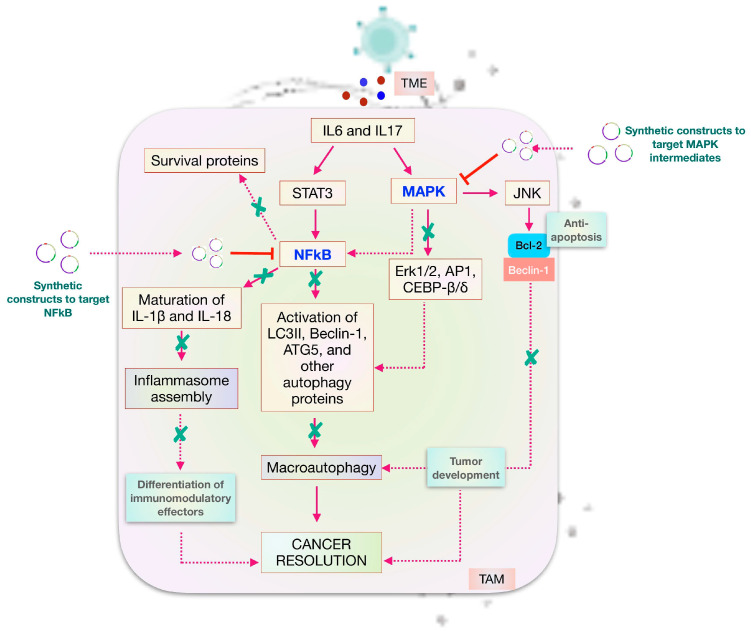
Regulation of NFkB and other signaling pathways to resolve cancer: IL-6 and IL-17 activate autophagy, progress inflammation through direct and indirect mechanisms, and result in cancer. Targeting NFkB and other signaling intermediates would restrict downstream effectors, thereby improving cancer outcomes.

Iterating through the results obtained as an effect of the IL-6/IL-17 signaling axis, it was really amazing to find the overexpression of those components that we really expected. At the end of an exercise, we found that NFkB is one of the crucial elements in the entire model that is activated through cell communication in almost all the cellular compartments as a result of IL-6 and IL-17. This led us to extend our findings to delve more into the biological intricacies. Interestingly, the higher concentration of NFkB signaled the assembly of an inflammasome complex and autophagy proteins that were expected to favor under-developed tumor mass toward uncontrolled proliferation. Analyzing the results, we may suspect that the upstream or downstream of NFkB drives cancer signaling in the TME. Therefore, to prove this, our study integrates in vitro and in vivo experiments in order to validate if these components are really cancer targets. And, establishing those data through wet lab experiments will be amazing and can be further translated into cancer patients for resolving lung inflammation (Figure 8).

## 4. Methodology

### 4.1. Data Collection

A thorough literature search was conducted to retrieve dispersed information from a biological database to elucidate the signaling network between the IL-6/IL-17 axis and autophagy [3,56].

### 4.2. Reconstruction of Mathematical Model for IL-6/IL-17 Axis and Autophagic Pathway

**Figure 9:**
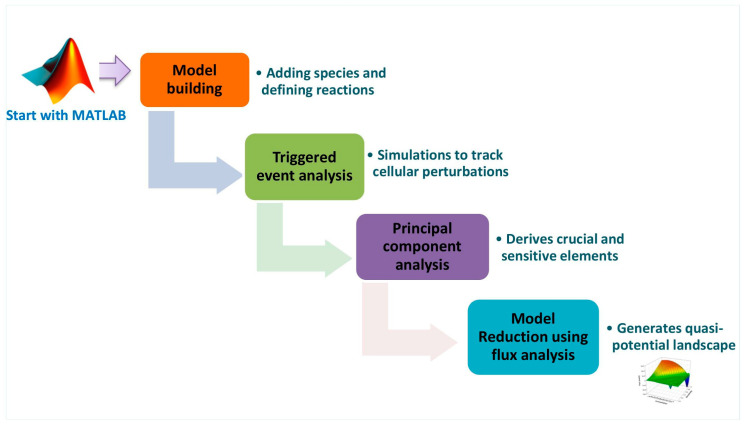
Workflow for the reconstruction of a mathematical model. The reconstruction of the mathematical model begins by defining specific rate laws and applying triggered events. The model is then simulated to explore the sensitive and principal elements in the reaction in order to obtain a dome-shaped graph.

Reconstructing a signaling network involves integrating data that outlines the biochemical changes of a particular network. It helps to obtain a greater understanding of how signaling proteins interact and provides an in-depth account of the complexities of biological systems. To fully comprehend fundamental intracellular and intercellular processes, quantitative modeling can be an approach to understanding these interactions [57]. In order to accomplish the goal, we used a computational approach for reconstructing the system’s immunological model to underline protein-protein interactions across a biological network. Given that perturbations in intracellular events are a result of environmental cues, these signaling axes were rebuilt in order to gain mechanistic insights regarding critical elements. In our proposed study, we reconstructed one mathematical model that represents chronic cancer inflammation in response to cytokine duality. This was achieved by employing the SBML-based MATLAB SimBiology Toolbox (v7.11.1.866) (The Mathworks Inc., Natick, MA, USA). Being a user-friendly tool, MATLAB allows users to manipulate reaction parameters and signaling molecule concentrations in order to produce a simulated model [58]. Figure 9 depicts the steps necessary to build a mathematical model.

Using the MATLAB^®^ software (v7.11.1.866), the model was built in a block diagram editor using compartments to indicate “cellular space”, species to represent “protein components”, and reactions to specify rate laws. For this purpose, the following rate laws were used to describe reactions in a model system: (1) The Law of Mass Action was used to explain association, dissociation, and translocation events; (2) Henri Michaelis Menten Kinetics was used to describe phosphorylation, dephosphorylation, and ubiquitination reactions; and (3) Hill Kinetics was used for gene expression-related reactions. The starting concentration of components was decided as per the literature, which states that an individual cell can release 10^3^–10^6^ signaling molecules [59]. As the model is built by mimicking the internal host environment, it helps establish the disease outcome by regulating the biochemical pathway. The network was computationally simulated for 100 time units using the SimBiology toolbox’s Stiff Deterministic ODE15s (stiff/NDF) solver, which creates first-order non-linear ODEs for each component to define model integrity [60]. Through simulations, the molecule concentration was examined and reduced in a range to avoid mathematical errors. The behavioral pattern of each system component was determined using a concentration vs. time graph [61].

### 4.3. Event Analysis

In MATLAB, an event refers to a sudden change in the species’ quantitative value under user-defined conditions. An event can only be triggered when the specified conditions in a given set of experiments are true, which furthers cellular transitions. In mathematical modeling, describing events is truly dependent on the intricate behavior of the model system, which can be either time-dependent or independent. Usually, events are prioritized to stimulate a particular reaction at a specific time point, with the aim of tracking immune perturbations. As a result of event analysis, the observed cellular changes in a model reflect similar conditions that can be identified in a host under the same settings. After model simulations, one can apply events to achieve the following major objectives: (1) to stimulate a particular reaction, (2) to inhibit any species or component of a model system, (3) to cause parametric and kinetic changes to remodel the model outcome, and (4) to mimic the biological host by adding specific stimulants to the modeled environment.

In order to understand the significance of applying triggered events and how they can offer an advantage in combating disease severity, we can consider a scenario of cancer inflammation, where the damaged pulmonary epithelium releases pro-inflammatory cytokines to heal the affected part. Since cancer is asymptomatic for early ages, once it starts spreading, it is difficult to stop and restrict at a specific site, which indicates that as the cancer progresses, there is an increase in the level of pro-inflammatory cytokines. This facilitates inflammatory events and produces anti-inflammatory cytokines to overcome an anti-tumor effect. Hence, at this stage, it becomes necessary to track if any of the cell subsets or host factors will be able to rescue the effects brought upon by cancer cells. To track such changes, we apply events that provide us with an idea of the cellular changes due to altered cytokine levels. Using this model and event analysis, we can, therefore, suspect the changes in the host system driven by any external stimuli (drug or antibody) to devise newer therapeutics for lung cancer.

For the study, we have used MATLAB (v7.11.1.866) to perform the triggered event analysis. In our model system, we applied events for a total of five reactions. While applying, we provided a specific and similar time for both the reactant and the product, which is “time ≥ 70 s”, and checked the inhibitory effect of one molecule on another subset to address cancer inflammation. The product concentration was filled to zero to simulate the particular reaction and to analyze the final value of the product. In the same way, we repeated the protocol for the other four reactions. The events were applied to inhibitory reactions where one molecule had an effect on another subset that decided the final outcome of the model. To apply events, we considered IL-6 to be a cytokine that aggravates cancer development at later stages, coinciding with the fact that IL-6 is pleiotropic in nature. Apart from IL-6, signaling components like GATA3, IL-10, IFNγ, and FoxP3 were also included in analyzing the model outcome. All these cytokines had a dominating effect on regulating the effector functions of immune types involved in lung cancer. Similar to IL-6, Th17 cells producing IL-17 were thought to have a tolerating effect during tumor development due to their pleiotropic nature. Immune cells like Treg cells were regarded as an immunomodulatory subset, whereas Th1 cells displayed an anti- tumor effect. Refer to Table 3 for a detailed triggered event analysis.

### 4.4. Sensitivity Analysis

Sensitivity analysis offers a significant understanding of the robustness of the model in response to external parameters and tells which model inputs are primary determinants of the model outcome. Though each component in a network is linked to specific reactions and rate laws, it aims to provide information on those components that are more affected and have an impact on biological systems. Therefore, sensitivity analysis may offer suggestions for how biological studies should be designed in order to get the most useful data.

In systems biology, there are two types of sensitivity analysis: local and global sensitivity analysis. We adopted local analysis to track the changes in model outcome with respect to small perturbations in the external environment [62]. The results were analyzed using a sensitivity coefficient, where all the elements were exposed to fitted parameters in order to calculate time-dependent sensitivities. The analysis was carried out using SUNDIALS as a default solver of the SimBiology toolbox (v7.11.1.866), which estimates sensitivity by incorporating initial ODEs with adjunct differential equations [59]. The sensitivity coefficient was calculated under a non-normalized configuration to avoid MATLAB changes in the reconstructed network. For a complete analysis, we considered a time plot to obtain positive values in the form of an *X*/*Y*-axis, where *X* denotes “time for 100 s” and *Y* indicates the sensitivity coefficient. The adjacency matrix was created using the digits obtained through the sensitivity graph and was further utilized to analyze PCs.

### 4.5. Principal Component Analysis

Often, large datasets are difficult to understand and correlate with their translational significance. To further explore big data analysis, reputed techniques are needed to simplify data while preserving its meaningful information. In order to serve this purpose, several tools have been designed, of which PCA is the widely accepted method that reduces data dimensionality to interpret biological networks [63]. Usually, it aims to discover crucial elements by separating dots that are of less importance. It can be employed to assess any data type, irrespective of the size of the samples, but the results typically vary between the sample type and its implementation.

Unlike other tools that offer a package for data analysis, one can expect PCA results to be more robust and reproducible. In disease biology, PCA has emerged as the oldest tool to be used for transcribing early datasets to figure out important biological entities connecting two different intracellular pathways. Toward the end, the reduction of multi- variate data into smaller dimensions provides PC [64]. Targeting those PCs would hamper the entire biological intricacies, which reflect that all derived components are equally important to drive disease conditions.

In Simbiology, the process briefly involves creating a transposed matrix of the elements that are produced through sensitive analysis. Finally, the PCA score can be calculated using the MATLAB function, which is >>[score_coefficient] = princomp(a), where a is the m*n matrix and the sensitivity coefficient score of one component with respect to other components in the entire network [61]. The derived coefficient table was imported into the SigmaPlot (15.0) in order to represent those components that are significant to the disease outcome. Using proper XY analysis, the graph was read to correlate inferences as per the expected disease outcome.

### 4.6. Flux Analysis

Flux analysis is a mathematical way to calculate the flow of metabolites in a given metabolic network and attempts to predict the highest concentration of immunologically important components across multiple reactions. It ensures determining the phenotype of a system by extracting more productive responses through mechanistic sorting of reactions based on higher flux. In a nutshell, adopting constraint analysis will derive the overexpressed components that determine the outcome of an entire model system [65].

For this purpose, COPASI (v4.35.258), a modeling simulator, was used to solve the ODE of an in-built model to dictate the final flux for each reaction. COPASI can support either of the experimental data types: time courses or steady-state measurements. For our study, the SBML format of a reconstructed network was executed under steady-state conditions in order to produce the time series data for the defined reactions.

### 4.7. Model Reduction

For many reasons, it may be desirable to limit the number of kinetic equations in a biochemical network so that the behavior of a few important metabolites can be well predicted [66]. For this purpose, we incorporated flux analysis and sensitivity analysis to formulate a systematic approach for removing reactions that do not considerably contribute to the network output. This is justified by the fact that reactions removed during the process may have both low flux and low sensitivity [67].

A mathematical approach was employed to numerically simulate the biological data to achieve the above-mentioned criteria. The main goal of MR in systems biology is the reduction of complex networks while preserving sensitive reactions [68]. In this study, the model reduction theory of chemical processes was used to explain reaction kinetics, whose derivation is based on a quasi-steady-state assumption. In order to establish the same findings, the MR data was procured using SBML file format, and the results were subjected to a SigmaPlot (15.0) so as to generate a 3D mesh graph bearing higher flux reactions.

### 4.8. Network Crosstalk Score

A crosstalk point refers to a direct or indirect interaction between signaling components of two different pathways that influence a biological response. In the context of the reconstructed network, these nodes tend to shape the model dynamics and have a key role in regulating signaling mechanisms [69]. Through analyzing the network’s topology, the process will help to discover the most crucial element, which implies that the higher the crosstalk score, the higher the chance that particular component facilitates inflammation.

Herein, the analysis was attempted for all cellular compartments to decipher the role of cytokines in triggering the autophagic and inflammatory events. For the purpose, we calculated a crosstalk between LPS-TLR4, IL-6/IL-6R, IL-17/IL-17R, IL-23/IL-23R, IFNγ-IFNγR, MCSF- MCSFR, IL-4/13-IL-4/13R, TGFβ-TGFβR, VEGF-VEGFR, PDGF-PDGFR, CCL2-CCR2, IL-1β/IL-1βR, IL-12/IL-12R, and TNFα-TNFαR. The analysis was conducted by subtracting the maximum degree of the node in each individual pathway from the total degree of the node in the entire network to elucidate the network’s regulatory components [70]. The “non-zero” value signifies that the analyzed elements are the crosstalk points in the network.

## 5. Future Perspectives

Earlier, the importance of IL-6 and IL-17 was more significantly noticed as pro-inflammatory molecules. However, with the increasing incidence of lung cancer and the pleiotropic nature of these cytokines, more studies are now focused on unraveling the crosstalk between the cytokines and their role in tumor development. Although the general roles of these cytokines have been investigated, their exact mechanistic regulation in progressing lung cancer is still undiscovered. This led to advancement in the study of cytokines and their related signaling axis in order to establish actionable nodes in NSCLC progression. As discussed, IL-6/IL-17-mediated activation of NFkB has a leading role in upregulating inflammasome factors and autophagic proteins in NSCLC. This is possible through the positive feedback mechanisms of IL-6 and IL-17 that cause cytokine storms and favor tumor-associated macrophages in the redox environment. What if we target the differentiation of anti-inflammatory subsets and attune immune responses for cancer resolution? This may really be a great approach to overcoming cancer survivability through modulating apoptotic mechanisms and limiting the production of angiogenic factors. In the revolutionized era of biology, this is achieved through computational approaches to designing synthetic constructs, drugs, or inhibitors for specific subsets or cytokines. By analyzing the model outcome through experimental data, the specificity of a peptide or drug can be used to improve the therapeutic potential of available chemotherapeutic drugs.

## Figures and Tables

**Table 1:** An overview of model characteristics involved in building mathematical model.

S. No	Model Characteristics	Total Parameters
1	Species	257
2	Reactions involved	175
3	Kinetic rate laws	335
4	Triggered events	5

**Table 2:** Flux analysis for the top ten reactions in a lung cancer model.

S. No.	Reactions	Flux (mol/s)
1	[T-CELL MEDIATED IMMUNE RESPONSE].[Th 17] -> [T-CELL MEDIATED IMMUNE RESPONSE].[IL -17A/F] + [T-CELL MEDIATED IMMUNE RESPONSE].[IL -6]	7542.65
2	[M2 MACROPHAGE].[Caspase 1] -> [M2MACROPHAGE].[IL-1b] + [M2MACROPHAGE].[IL-18]	7458.54
3	[T-CELL MEDIATED IMMUNE RESPONSE].[IL-17A/F] -> [TYPE III IMMUNE RESPONSE].[IL-17 A/F] + [MONOCYTE-TO-M1 POLARIZATION].[Mo_IL-17 A/F] + [M2 MACROPHAGE].[M2_IL-17A/F]	6885.11
4	[M2 MACROPHAGE].[Nlrp3 complex] -> [M2 MACROPHAGE].[Capase 1]	6748.76
5	[M2 MACROPHAGE].[Nemo complex] -> [M2 MACROPHAGE].M2_NFkB	5357.28
6	[TYPE III IMMUNE RESPONSE].[Nemo complex] -> [TYPE III IMMUNE RESPONSE].NFkB	5155.21
7	[T-CELL MEDIATED IMMUNE RESPONSE].[STAT 3] + [T-CELL MEDIATED IMMUNE RESPONSE].[RORgt gene] -> [T-CELL MEDIATED IMMUNE RESPONSE].RORgt + [T-CELL MEDIATED IMMUNE RESPONSE].[STAT 3]	5078.57
8	[M2 MACROPHAGE].M2_NFkB + [M2 MACROPHAGE].[Nlrp3 gene] -> [M2 MACROPHAGE].Nlrp3 + [M2 MACROPHAGE].M2_NFkB	4979.32
9	[M2 MACROPHAGE].PI3k -> [M2 MACROPHAGE].Akt	4951.11
10	[TYPE III IMMUNE RESPONSE].[IL-4/13] -> [T-CELL MEDIATED IMMUNE RESPONSE].[IL- 4/IL- 13] + [M2 MACROPHAGE].[IL-4/IL13] + MDSC.[IL-4/IL-13]	4885.72

**Table 3:** Triggered event analysis in lung cancer model: IL-6, IFNγ, and FoxP3 regulated the activity of Treg and Th17 cells, respectively, to have an anti-tumor effect upon cancer transformation. In contrast, transcription factor GATA3 and cytokine IL-10 inhibited Th1 cells to generate a tolerating effect in rising inflammation. The events were applied to check the effect of different effector molecules on the host’s immune environment, which can be further targeted to achieve cancer immunotherapy.

S. No.	Effector Molecules	Modulated Subset	Model Outcome
1	IL-6	Treg	Immune Response
2	GATA3	Th1 phenotype	Immune Tolerance
3	IL-10	Th1 phenotype	Immune Tolerance
4	IFNγ	Th17 cell	Immune Response
5	FoxP3	Th17 cell	Immune Response

## Data Availability

The data that support the findings of this study are available in the Supplementary Materials.

## Supplementary Materials

The following supporting information can be downloaded at https://www.mdpi.com/article/10.3390/ijms25021216/s1.

## Abbreviations

AP1	Activating Protein 1
BSF-2	B-cell Stimulating Factor-2
CAF	Cancer-Associated Fibroblast
CEBP-β/δ	CCAAT/enhancer-binding protein beta/delta
CCL2	Chemokine (C-C motif) ligand 2
CCR2	C-C chemokine receptor type 2
DC	Dendritic Cell
EMT	Epithelial-to-Mesenchymal Transition
Erk1/2	Extracellular signal-regulated protein kinases
*Hif-1α*	Hypoxia-inducible factor-1α
HIV	Human Immunodeficiency Virus
IFNγ	Interferon-gamma
IFNγR	Interferon-gamma Receptor
IL	Interleukin
iNOS	inducible Nitric Oxide Synthase
JNK	Janus Kinase
LPS	Lipopolysaccharide
MAPK	Mitogen-Activated Protein Kinase
MATLAB	MATrix LABoratory
MCSF	Macrophage Colony-Stimulating Factor
MCSFR	Macrophage Colony-Stimulating Factor Receptor
MDSC	Myeloid-Derived suppressor Cell
Mo	Monocytes
MR	Model Reduction
Myd88	Myeloid Differentiation primary response 88
NDF	Numerical Differentiation Formula
NFkB	Nuclear Factor kappa B
NK	Natural Killer
NLRP3	Nucleotide-binding domain, leucine-rich containing family, pyrin domain– containing-3
NSCLC	Non-Small Cell Lung Cancer
ODE	Ordinary Differential Equation
PC	Principal Component
PCA	Principal Component Analysis
PDGF	Platelet-Derived Growth Factor
PDGFR	Platelet-Derived Growth Factor Receptor
PIC	Pro-Inflammatory Cytokine gene
PSM	Proliferation, Survival, and Migration
RORγt	RAR-related orphan receptor gamma t
SBML	Systems Biology Markup Language
TAM	Tumor-associated Macrophages
TC	Tumor Cell
TGFβ	Transforming Growth Factor-beta
TGFβR	Transforming Growth Factor-beta Receptor
Th	T-helper
TME	Tumor microenvironment
TNFα	Tumor necrosis factor- alpha
TNFαR	Tumor necrosis factor- alpha Receptor
TLR	Toll-like Receptor
Treg	T-regulatory cell
VEGF	Vascular Endothelial Growth Factor
VEGFR	Vascular Endothelial Growth Factor Receptor
